# Heterogeneity in Metabolic Responses to Dietary Fructose

**DOI:** 10.3389/fgene.2019.00945

**Published:** 2019-10-31

**Authors:** Ruixue Hou, Chinmayee Panda, V. Saroja Voruganti

**Affiliations:** Department of Nutrition and UNC Nutrition Research Institute, University of North Carolina at Chapel Hill, Kannapolis, NC, United States

**Keywords:** genetic variants, complex diseases, metabolic response, added sugars, individual variability

## Abstract

Consumption of fructose has dramatically increased in past few decades in children and adults. Increasing evidence indicates that added sugars (particularly fructose) have adverse effects on metabolism and lead to numerous cardiometabolic diseases. Although both fructose and glucose are components of sucrose and high fructose corn syrup, the sugars have different metabolic fates in the human body and the effects of fructose on health are thought to be more adverse than glucose. Studies have also shown that the metabolic effects of fructose differ between individuals based on their genetic background, as individuals with specific SNPs and risk alleles seem to be more susceptible to the adverse metabolic effects of fructose. The current review discusses the metabolic effects of fructose on key complex diseases and discusses the heterogeneity in metabolic responses to dietary fructose in humans.

## Introduction

Increasing evidence indicates that added sugars (particularly fructose) have adverse effects on metabolism leading to diseases such as obesity, hyperuricemia, hypertension, gout, type 2 diabetes (T2D), non-alcoholic fatty liver disease (NAFLD), and cardiovascular diseases (CVD) (Vos et al., 2008; Lim et al., 2010; Aeberli et al., 2011; Herman and Samuel, 2016; Softic et al., 2016; Hannou et al., 2018). Consumption of fructose has dramatically increased in past few decades (Vos et al., 2008; Marriott et al., 2009). Americans consumed about 15 g/day (60 calories) fructose mainly from fruits and vegetables before 1900, about 24 g/day before World War II, 37 g/day by 1977, and 55 g/day by 1994 (Softic et al., 2016). In 2004, an average of 50 g/day (10% of calories) consumed were attributed to fructose. Among the age groups, adolescents (12–18 years) consumed the most fructose, more than 72.8 g/day (∼290 calories) of fructose (Vos et al., 2008; Lim et al., 2010). Notably, a recent scientific statement from the American Heart Association (AHA) (Johnson et al., 2009) recommended consuming ≤ 100 calories for women and children over 2 years of age, and ≤ 150 calories for men from added sugars *per*day to avoid the detrimental metabolic effects of added sugars.

Fructose, commonly known as fruit sugar, is a simple monosaccharide with a 6-carbon polyhydroxyketone backbone and makes up 50% of the composition of sucrose. While natural sources of fructose include fruits, root vegetables, and honey, industrial sources use a highly processed and concentrated form of crystalline fructose for production of sugar-sweetened beverages (SSBs) and energy drinks (Hanover and White, 1993; Klurfeld et al., 2016). Compared to glucose, the low cost and high relative sweetness of fructose (as in high fructose corn syrup-HCFS) makes it a commercial favorite for imparting added sweetness and flavor to processed foods and beverages (Hanover and White, 1993; Zosia, 2012; Klurfeld et al., 2016).

**Figure 1:**
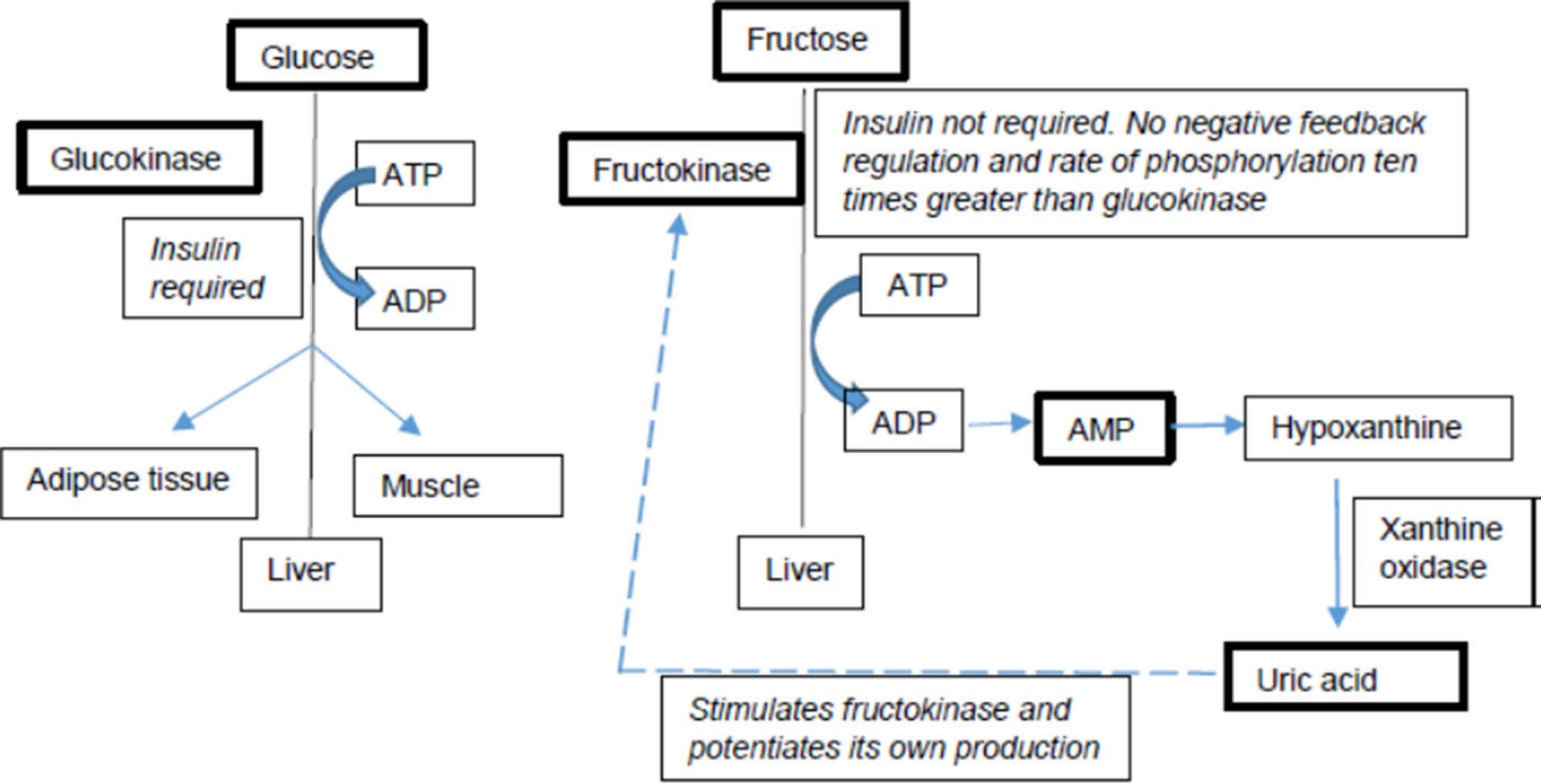
Differences in fructose vs. glucose metabolism. Key players are bolded.

Fructose and glucose have different metabolic fates in the human body (**Figure 1**), in particular with respect to hepatic metabolism (Mayes, 1993; Basciano et al., 2005; Sun and Empie, 2012). Additionally, multiple studies have also shown that the metabolic effects of fructose differ between individuals based on their genetic background. Individuals with specific single nucleotide polymorphisms (SNPs) and risk alleles seem to be more susceptible to the adverse metabolic effects of fructose than those without them (Goran et al., 2012; Dalbeth et al., 2013; Dalbeth et al., 2014a; Dalbeth et al., 2014b; Kong et al., 2017). Several genetic studies provide evidence that individuals not only differ in their fructose and glucose metabolism but also differ in their metabolic response to fructose based on their genetic variations. These differences are particularly pertinent in light of the recent AHA sugar intake recommendations. This review discusses the metabolic effects of fructose on key risk factors of complex diseases and discusses the heterogeneity in metabolic responses to dietary fructose in humans.

## Obesity/Adiposity

Dietary fructose seems to increase the risk for obesity both at central as well as peripheral levels. At a central level, fructose affects appetite by upregulating hypothalamic cannabinoid mRNA and decreasing the activity of brain satiety centers whereas at the peripheral level it modulates the concentrations of ghrelin and leptin (Lindqvist et al., 2008; Lowette et al., 2015). Additionally, SSB and fructose consumptions have been positively associated with higher caloric intake, which contributes to increased adiposity (Tappy and Le, 2010). Several studies and meta-analyses have reported positive relationships between fructose intake and obesity in children, adolescents, and adults (Johnson et al., 2007; Forshee et al., 2007; Tappy and Le, 2010). Berkey et al. (2004) showed that every increase in sugar serving/d was associated with an increase of 0.16 units of BMI. A study in two large Swedish cohorts with more than 26,000 participants found that SSB consumption was positively associated with BMI (Brunkwall et al., 2016). SSB intake was categorized into quartiles and each successive category was associated with 0.18 units increase in BMI (Brunkwall et al., 2016). However, several studies included in the meta-analysis in adults or children reported no difference in BMI based on SSB consumption (Libuda et al., 2008; Vanselow et al., 2009; Reid et al., 2010; Malik et al., 2013). These conflicting findings between studies regarding the relationship between SSB intake and BMI could be attributed to different population characteristics including age, gender, physical activity, ethnicity, and genetic variation, or study design differences like exposure assessments and covariates adjustment in data analysis.

The genetic contribution to obesity has been extensively studied and several loci with significant effects have been identified (Qi and Cho, 2008; Speliotes et al., 2010; Brunkwall et al., 2016; Olsen et al., 2016). However, the genetic loci account for a small proportion of heritability, pointing to the role of gene × environment interactions in the variation in obesity phenotypes (Heianza and Qi, 2017). For example, the POUNDS Lost trial has identified heterogeneity in body fat composition and central adiposity response to SSB/fructose intake that includes interaction effects of rs838147 at fibroblast growth factor 21 (*FGF21*) gene with carbohydrate/fat consumption (Heianza et al., 2016). The authors found that rs838147 C allele carriers had a greater reduction in waist circumference, total body fat, and trunk fat as compared to other allele carriers (Heianza et al., 2016).

Since individual SNPs may not explain a large proportion of the variance in a phenotype, some studies have investigated the cumulative effects of multiple SNPs using genetic risk scores (GRSs). A GRS is calculated by adding the number of alleles in significant SNPs, either unweighted or weighted by their effect sizes with the goal of improving risk prediction. In two large Swedish cohorts (Brunkwall et al., 2016), a GRS was computed using 30 genetic loci associated with BMI. They found that the association of BMI with SSB intake was stronger in individuals who had a higher GRS (i.e. genetically predisposed to obesity) than those with a lower GRS, which is consistent with another study in the US (Qi et al., 2012). Similarly, a Finnish study has shown that there was a stronger association between SSBs and weight gain in people with higher GRS for high BMI. Interestingly, this study also found that there was an attenuated association between SSBs and weight gain in people with a genetic predisposition to high waist circumference (Olsen et al., 2016). This may be explained by the differences in body fat distribution. BMI reflects the overall fatness and does not differentiate lean mass from fat mass, whereas waist circumference is more indicative of abdominal fat (Staiano et al., 2012). Overall, it is becoming evident that genetic differences in metabolism can have a substantial impact on energy balance.

## Type 2 Diabetes

As of 2015, about 9% of Americans (30.3 million adults and children) had diabetes, of which 29 million had type 2 diabetes (T2D) (http://www.diabetes.org/diabetes-basics/statistics/). Like many other complex diseases, diabetes is a multifactorial disease affected by genes, environment (mainly diet), and gene–environment interaction (Ortega et al., 2017). Fructose was initially thought to be an appropriate substitute for glucose in diabetic patients as it does not stimulate insulin secretion. However, chronic fructose feeding seems to cause insulin resistance and results in higher plasma insulin (Basciano et al., 2005; Teff et al., 2009; Tappy and Le, 2010). Similar results were obtained from a meta-analysis on intervention trials with respect to HbA1c when fructose intake was restricted to < 90 g/day in adults (Livesey and Taylor, 2008). Stanhope et al. (2009) found that fructose, but not glucose, supplementation for 10 weeks increased fasting glucose and insulin concentrations and decreased insulin sensitivity. Additionally, a meta-analysis found that higher SSB intake by one serving *per*day was associated with a greater incidence of type 2 diabetes by 18% and 13% before and after adjustment for adiposity, respectively (Imamura et al., 2016). However, study results have been controversial with respect to effects of fructose on diabetes. A systematic review of 18 feeding trials found that the isocaloric exchange of fructose with other carbohydrates had no effect on circulating glucose and insulin concentrations (Cozma et al., 2012). Similar result was found in an intervention study in adults where adults were given either low-fat unsweetened milk or low-fat milk sweetened with fructose or low-fat milk sweetened with glucose for 10 weeks (Lowndes et al., 2015).

The search for genes affecting T2D and/or related risk factors has extended for more than two decades. Key genes that have been reported to be associated with T2D-related phenotypes are transcription factor family 7, member 2 (*TCF7L2*), peroxisome proliferator-activated receptor gamma (*PPARG*), and potassium voltage-gated channel subfamily J, member 11 (*KCNJ11*) (Franks, 2012). Candidate gene association studies have shown that SNPs in solute carrier family 2, member 2 (*SLC2A2*) are associated with beta-cell function, insulin action and higher risk of developing type 2 diabetes (Barroso et al., 2003; Laukkanen et al., 2005; Willer et al., 2007). Since both glucose and fructose are transported by SLC2A2, both glucose homeostasis and fructose homeostasis could be affected by *SLC2A2*polymorphisms, which may magnify the development of adverse health outcomes. Moderate to vigorous physical activity has been found to modify the association of *SLC2A2*with glucose levels and the conversion from impaired glucose tolerance to type 2 diabetes (Kilpeläinen et al., 2007). Given the association of fibroblast growth factor 21 (*FGF21*) variants with adiposity and glucose metabolism, a recent meta-analysis analyzed 11 genes in fructose metabolism and carbohydrate response element-binding protein (ChREBP)-FGF21 pathways (McKeown et al., 2018). They found that SSB intake was associated with higher fasting glucose and insulin. However, they did not find any interaction effects of SNPs in these genes and SSB intake on glycemic traits (McKeown et al., 2018). Instead, they found only a suggestive interaction of a SNP in the beta-Klotho (*KLB*) locus with SSB intake on fasting insulin in the discovery cohort that was not replicated in replication cohorts or meta-analysis (McKeown et al., 2018).

## NAFLD and Dyslipidemia

Fructose is unique, and distinct from glucose, in its effects with respect to NAFLD or dyslipidemia (Vos and Lavine, 2013). The rate of phosphorylation of fructose by ketohexokinase (KHK) is 10 times higher than the phosphorylation of glucose by glucokinase and, in turn, fructose stimulates KHK and accelerates its own metabolism. Glucose does not increase *de novo*lipogenesis (DNL) at the same rate and magnitude of fructose. Fructose is also directly absorbed into portal vein and delivered to the liver without entering the systemic circulation, which exposes liver to a much higher fructose load than other tissues. In addition, fructose activates the lipogenic transcriptional factors, sterol regulatory element-binding protein 1c (SREBP1c) and ChREBP in the liver to promote DNL. Phosphorylation of fructose depletes liver ATP levels with a consequence of increased AMP, which is converted to urate*via* the purine degradation pathway. Urate has been shown to upregulate KHK and stimulate fat synthesis in the hepatocyte, pointing to an additional pathway through which fructose can induce liver lipogenesis (Stanhope, 2016; Ter Horst and Serlie, 2017; Chiu et al., 2018).

Fructose overconsumption stimulates lipogenesis, contributing to an increase in triglyceride levels and steatosis (Herman and Samuel, 2016; Hannou et al., 2018). Numerous studies have demonstrated that diets high in simple sugars including fructose may result in elevated fasting triglycerides (TGs) or hypertriglyceridemia, often accompanied by a decrease in high-density lipoprotein (HDL) cholesterol (Eckel et al., 2005; Bantle, 2009). A meta-analysis has also found a positive relationship between fructose intake and triglyceride levels (Livesey and Taylor, 2008). Increased hepatic TG synthesis, DNL in the liver and reduced peripheral TG clearance all have been attributed to increased fructose metabolism (Zhang et al., 2013a; Stanhope, 2016). A recent meta-analysis has reported that only hypercaloric but not isocaloric substitution of fructose for other carbohydrate sources, was associated with elevated fasting low-density lipoprotein (LDL) cholesterol and TGs or postprandial TGs (Zhang et al., 2013a). In contrast, a randomized controlled trial, which investigated the effect of 8, 18, and 30% of calories from SSBs, corresponding to 25, 50, and 90^th^ percentile of population consumption of fructose, respectively (Lowndes et al., 2014), observed no change in LDL cholesterol and only minimal changes in body weight and circulating levels of HDL cholesterol and TGs. Thus, the effects of fructose on LDL cholesterol remain debatable. Clearly, more trials reflecting normally consumed levels of fructose-containing sugars in the population are warranted. Moreover, several studies have shown that fructose affects lipogenesis in liver in a genotype-specific manner. Davis et al. (2010) found that Hispanic children with GG genotype of Patatin Like Phospholipase Domain Containing 3 (*PNPLA3*) SNP rs738409 were more inclined to accumulate fat in the liver with a diet of high sugar consumption or dietary carbohydrate intake as compared to children with CC or CG genotypes. Similarly, a group of subjects recruited based on their GG or CC genotypes of *PNPLA3*SNP, rs738409, had differential responses to a low-carbohydrate hypocaloric diet. Although both GG and CC subjects had similar weight loss, GG subjects lost more hepatic fat (45%) than CC subjects (18%) (Sevastianova et al., 2011). In yet another study investigating the effects of sugars (oral glucose + fructose challenge) found that individuals with TT genotype of rs1260326 of glucokinase regulator (*GCKR*) demonstrated higher fractional DNL and a lower increase in fractional DNL as compared to CC subjects (Santoro et al., 2015).

## Hypertension

While high sugar intake in general is associated with development of obesity and hypertension, fructose appears to have an independent effect on pathogenesis of these diseases (Jalal et al., 2010). Epidemiological observations and experimental evidence from animal and human studies have reported an association between high fructose consumption and development of hypertension (Johnson et al., 2007; Brown et al., 2008; Madero et al., 2011). In US adolescents, SSB intake was positively associated with urate levels and blood pressure (Nguyen et al., 2009). Similarly, results were observed in adults where an increase of one serving of SSB was associated with an increase of ∼2 mm Hg of systolic (SBP) and diastolic (DBP) blood pressure (Brown et al., 2011). A 1.8 mm Hg reduction in SBP and a 1.1 mm Hg reduction in DBP were also observed in US adults, who were administered one less SSB *per*day over a period of 18 months (Chen et al., 2010). Another study found in 7 healthy young adults that ingestion of fructose increases BP to a greater extent than glucose or sucrose ingestion (Grasser et al., 2014). Animal studies investigating the mechanisms of fructose-induced hypertension have shown that high-fructose diets increase blood pressure through a state of salt overload *via*up-regulation of sodium and chloride transporters, activation of vasoconstrictors, inactivation of vasodilators, and over-stimulation of the sympathetic nervous system (Wong et al., 2016).

Investigations of the relationship between SSB intake and metabolic responses have also shown a statistically significant rise in blood pressure with fructose ingestion in healthy adults (Cai et al., 2018). Similar findings are also corroborated in fructose-fed animal studies (Nakagawa et al., 2006; Crescenzo et al., 2018). According to Johnson et al. (2007), fructose-induced hyperuricemia may contribute to endothelial dysfunction and increased risk of hypertension. However, other studies have not found increase in blood pressure or urate with added sugar consumption levels up to 30% of Cal/d which is at about the 95th percentile population consumption level of fructose (Rippe and Angelopolous, 2015). In another randomized trial, administration of fructose, sucrose, or HFCS at average population consumption level (50th percentile) did not raise clinically significant blood pressure or urate when compared with a glucose control (Angelopoulos et al., 2016). Thus, more studies are required to understand the effects of intake of simple sugars on hypertension at average population levels.

## Hyperuricemia and Gout

Another distinction between fructose and glucose is that the metabolism of fructose results in increased serum urate concentration (Kang and Ha, 2014). This is mediated by the activity of KHK which is distinct from other hexokinases in its ability to induce transient adenosine triphosphate (ATP) depletion in the cell as a consequence of its rapid phosphorylation of fructose to fructose-1-phosphate. Since the majority of fructose metabolism occurs in the liver, this ATP depletion impacts other hepatic metabolic processes. The depletion in ATP leads to intracellular phosphate depletion and consequently a dramatic increase in AMP levels. This phenomenon stimulates catabolic activity of the enzyme AMP deaminase resulting in the ultimate degradation of AMP to urate. Urate is thus the end product of purine nucleotide catabolism and hyperuricemia is characterized by excess production and deposition of urate crystals leading to painful gout. Clinical trial conducted by Cox et al. (2012) also found that fructose-sweetened beverages (25% of energy requirement) for 10 weeks, compared to glucose-sweetened beverages, led to significant increases in 24-h uric acid. Excess consumption of HFCS can exacerbate this condition (Sanchez-Lozada et al., 2007; Jamnik et al., 2016; Kanbay et al., 2016). The relationship between fructose metabolism-mediated hyperuricemia and development of the metabolic syndrome features including obesity, visceral fat accumulation, fatty liver, and elevated insulin and leptin levels has been demonstrated in fructose-fed animals (Nakagawa et al., 2006; Crescenzo et al., 2018). This fructose-induced metabolic syndrome was also shown to be reduced by treating animals with allopurinol, a urate lowering drug (Rippe and Angelopoulos, 2016). Similar observations were reported in adult men by Perez-Pozo et al. (2010) where increase in blood pressure due to high dose of fructose was attenuated by allopurinol. It is also important to note that cellular release of fructose metabolites like urate can result in detrimental histopathological changes in some organs under high fructose conditions (Zhang et al., 2017). For example, high serum urate has been reported to induce reactive oxygen species (ROS) production, lipid accumulation, autophagy, and inflammatory cytokine flux while reducing sensitivity to insulin and leptin in brain, adipose tissue, and kidney through the inflammatory response and endothelial dysfunction. In heart, these metabolites additionally trigger vascular vasodilation and hypertension (Li et al., 2016). Urate is also known to increase intestinal permeability through induction of endotoxin translocation and disruption in bacterial composition (Kayhan et al., 2008). Additionally, urate is known to mediate urine sodium retention, dysregulate renal organic ion transporters, and nitric oxide production in chronic kidney disease (CKD) (Johnson et al., 2013).

SSB consumption is also associated with hyperuricemia-associated disease, gout (Choi and Curhan, 2008; Choi et al., 2008). The key urate transporter, solute carrier channel family 2, member 9 (SLC2A9), transports both fructose and urate and fructose might interfere with urate transport (Witkowska et al., 2012). Genetic variations of *SLC2A9*may explain up to 1 to 2% of urate variation in males and 5 to 6% in females (Le et al., 2008). Genetic studies have found that *SLC2A9*SNPs were strongly associated with urate levels in various populations and that there are gender-specific effects (Brandstätter et al., 2008; Dehghan et al., 2008; Wallace et al., 2008; Merriman, 2015; Voruganti et al., 2013; Voruganti et al., 2014; Voruganti et al., 2015; Merriman, 2017). In our own studies, we found that minor alleles of key SNPs in *SLC2A9*and ATP binding cassette subfamily G, member 2 (*ABCG2*), another key urate transporter were associated with lower serum urate concentrations (Voruganti et al., 2013; Zhang et al., 2013b; Voruganti et al., 2014; Voruganti et al., 2015). Clinical trials have also shown that acute serum urate and fractional excretion of urate responses to a fructose load were influenced by *SLC2A9*genotypes (Dalbeth et al., 2013). Since fructose plays a major role in the production and excretion of urate, studies have investigated the interaction effects of SSB/fructose with urate transporter SNPs on serum concentrations of urate. An observational study has also found a non-additive interaction effect between SSB consumption and *SLC2A9*genotypes on gout risk, suggesting that simple sugar exposure from SSB consumption could affect the ability of SLC2A9 to transport urate (Batt et al., 2014). Clinical trials also found that *SLC17A1*and *ABCG2*genotype influence serum urate and fractional excretion of urate following a fructose load in two different populations, Europeans and New Zealand Maoris, respectively (Dalbeth et al., 2014a; Dalbeth et al., 2014b).

## Rare Genetic Mutations and Response to Fructose Intake

The main disorders of fructose metabolism caused by rare genetic mutations are essential fructosuria, hereditary fructose intolerance, and fructose-1, 6-bisphosphatase deficiency.

### Essential Fructosuria

Essential fructosuria is a harmless, asymptomatic disorder characterized by intermittent appearance of fructose in the urine. It is caused by a deficiency of KHK, the first enzyme of main fructose metabolism pathway, and the mode of inheritance is autosomal recessive. In a well-characterized family with fructosuria, two mutations in the *KHK*gene, G40R and A43T, were found to alter the same conserved region of the KHK protein (Bonthron et al., 1994). Ingestion of dietary fructose in people with essential fructosuria is followed by an increase in blood fructose concentration and excretion in the urine (Gitzelmann et al., 1989). Fructosuria depends on the time and amount of sucrose and fructose intake.

### Hereditary Fructose Intolerance

Individuals with hereditary fructose intolerance (HFI) may develop severe liver and kidney failure and death with fructose exposure. It is caused by a deficiency of aldolase B, the second enzyme of the fructose pathway, resulting from homozygous or compound heterozygous mutations in the *ALDOB*gene (Gitzelmann et al., 1989; Esposito et al., 2002). Heterozygous carriers have about 50% of *ALDOB*activity, which is presumed to be sufficient for individuals to have normal fructose metabolism. However, studies have found an increase in urate concentration in response to modest fructose ingestion even though no significant differences in fructose metabolism were reported (Oberhaensli et al., 1987; Beosiger et al., 1994; Debray et al., 2018). The avoidance of fructose, sucrose and/or sorbitol from the diet is the major therapeutic step in HFI and most abnormalities disappear with a fructose-free diet except for hepatomegaly with unclear reasons (Odièvre et al., 1978; Gitzelmann et al., 1989).

### Fructose-1, 6-Bisphosphatase Deficiency

Fructose-1, 6-bisphosphatase (FBP1) deficiency is characterized by impaired gluconeogenesis (Gitzelmann et al., 1989). It usually presents in the newborn period with profound metabolic acidosis and hypoglycemia. Episodes of hyperventilation, irritability, coma, and ketosis may occur later on (Baker and Winegrad, 1970; Kikawa et al., 1997; Matsuura et al., 2002). It is caused by mutation in gene *FBP1*. There are also several cases with no mutations found within *FBP1*and it is hypothesized that these patients have mutations within the *FBP1*promoter region (Hers and Van Schaftingen, 1982; Gitzelmann et al., 1989). Adequate amounts of glucose should be given if this disorder is suspected and the course is usually benign with proper management (Gitzelmann et al., 1989).

Besides these three main fructose disorders, *SLC2A2*mutations could result in impaired glucose and galactose utilization and *SI*defects can cause sucrase–isomaltase deficiency due to abnormal sucrose breakdown (Gray et al., 1976; Manz et al., 1987; Santer et al., 1997). In addition to the rare genetic mutations that influence fructose metabolism, common polymorphisms can also affect inter-individual variability in fructose metabolism and related adverse health effects.

## Differential Expression of Genes in Response to Dietary Fructose

Dietary fructose is also known to contribute to metabolic deterioration through its effects on expression of genes involved in the disease pathways. A transcriptomic analysis in health young offspring of T2D patients given a high fructose diet for 7 days showed significant upregulation of genes implicated in energy and lipid metabolism (Seyssel et al., 2016). In another study of 5 healthy adults, transcriptomic analysis of skeletal muscle showed increased expression of stearoyl-CoA desaturase 1 (SCD1) by 50% and reduced the expression of glucose transporter 4 (GLUT4) by 27% and acetyl-coA-carboxylase by 48% indicating a trend towards development of insulin resistance (Le K. A., et al., 2008). Similar results have been observed in animal studies in which dietary fructose causes perturbation in expression of genes associated with cardiometabolic diseases (Nagai et al., 2009; Meng et al., 2016; Stamatikos et al., 2016). In another study in rats, maternal fructose consumption was associated with detrimental effects on metabolism in offsprings, some of which may persist into adult life (Tain et al., 2016).

## Summary

In this review, we have described the metabolic effects of dietary fructose on risk factors of complex diseases and the heterogeneity in metabolic responses to dietary fructose. To a large extent, the differential response in metabolic disease risk factors to dietary fructose seems to depend on the genetic background. Individuals with specific SNPs and risk alleles are more susceptible to the adverse metabolic effects of fructose than those without them. Thus, to recommend a safe threshold of fructose consumption, it is necessary to understand the heterogeneity in metabolic responses. Limited studies investigated the interactions between gene and fructose (**Table 1**) of which majority are yet to be replicated.

**Table 1:** Summary of fructose × gene interaction effects on cardiometabolic disease risk factors.

Disease/risk factor	Phenotype	Study type	Ethnicities	N	Diet/nutrient intake	SNP/GRS	Outcome	Reference
Obesity	Weight gain and waist circumference (WC)	Cohort	Caucasians	4765	Soft drinks	GRS of 50 SNPs	Genetic susceptibility to a high WC may attenuate the association between soft drink intake and weight gain, while genetic predisposition to high BMI may strengthen the association between soft drink intake and WC gain.	Olsen et al. (2016)
	BMI	Cohort	Caucasians	33,097	SSB	GRS of 32 BMI associated-SNPs	Genetic association with BMI was more pronounced in people with higher SSB intake.	Qi et al. (2012)
Type 2 diabetes	Fasting glucose and fasting insulin	Cohort	Caucasians	34,748	SSB	ChREBP-FGF21 pathway SNPs	No statistically significant interaction effects were found between SSB and ChREBP-FGF21 pathway SNPs on fasting glucose and fasting insulin.	McKeown et al. (2018)
NAFLD/ Dyslipidemia	Liver fat	Clinical trial	Hispanics	153	Total sugar	rs738409 (*PNPLA3*)	GG carriers were more susceptible to hepatic fat accumulation when dietary sugar intake is high.	Davis et al. (2010)
	Liver fat	Clinical trial	Finnish	18	Hypocaloric low-carbohydrate diet	rs738409 (*PNPLA3*)	Weight loss is effective in decreasing liver fat by 45% in GG subjects and 18% in CC subjects.	Sevastianova et al. (2011)
Hyperuricemia/ Gout	Serum urate and gout	Case-control	Caucasians/New Zealand Maoris/Pacific Islanders	1,634	SSB	rs6449173, rs11942223 (*SLC2A9*)	SLC2A9-mediated urate excretion was influenced by intake of SSB.	Batt et al. (2014)
	Serum urate excretion and hyperuricaemic response	Clinical trial	Caucasians/Maoris/Pacific (Western Polynesian)	76	Fructose	rs11942223 (*SLC2A9*)	*SLC2A9*variation affected urate excretion and hyperuricaemic response to fructose intake.	Dalbeth et al. (2013)

The concept of nutrigenetics is not entirely new, given the classic examples of phenylketonuria and lactase persistence. However, these two diseases are monogenic unlike most common diseases which are complex and involve multiple genes, gene–gene, and gene–environment interactions. Recent advances in nutrigenetics have provided scientific evidence for effects of gene × nutrient interactions with respect to few risk factors of complex diseases. For example, many studies have shown the effects of interaction between methylenetetrahydrofolate reductase (*MTHFR*) SNP rs1801133 (C677T) and dietary folate to affect homocysteine levels with implications for CVD (Papoutsakis et al., 2005; Nishio et al., 2008; Liew and Gupta, 2015). With more studies that involve standardized measures of dietary intake, comprehensive set of validated genetic variants and “omics” approaches beyond genotyping, we will be able to achieve a greater understanding of the individual variability in metabolic responses to fructose.

## Author Contributions

RH, CP, and VSV wrote the manuscript. VSV designed the study.

## Funding

This study was partly funded by NIH/NIDDK- DK056350 to VSV.

## Conflict of Interest

The authors declare that the research was conducted in the absence of any commercial or financial relationships that could be construed as a potential conflict of interest.

The handling editor declared a past collaboration with one of the authors VSV.
